# Supplementary material for: Experiences of Veterans, Caregivers, and VA Home-Based Care Providers before, during, and Post-Hurricane Ian
Source: Geriatrics (Basel). 2024 Jan 10;9(1):10. doi: 10.3390/geriatrics9010010 (PMC10801461; doi:10.3390/geriatrics9010010)
Supplement: Supplementary file 1 [file geriatrics-09-00010-s001.zip › Interview Guide S1 Veterans.pdf]

Interview Guide for VA Veterans receiving Home Based Long-Term Care or Medical Foster Home Care Before, During, After Hurricane Ian

1. Tell me about what types of care you receive from the VA.

   a. *Probes*
      i. Tell me more about what it is like receiving that care (specifically probe on whether they receive care from the VA Medical Foster Home program, or the VA Home Based Primary Care program, or other VA long-term services and supports they may get, like home health)

      ii. Do you receive most of your healthcare appointments at home or in the clinic?
         a. If in the clinic, how do you get to your healthcare appointments?
         b. Does anyone help with your care, getting to your appointments, or your medications?

   b. How long have you received care from the VA?

      i. How long have you received care from the specific programs you mentioned (i.e. MFH program or the HBPC program or home health)?

      ii. How did you decide to receive these types of care (i.e. from MFH or HBPC or home health, etc.)?

   c. How do you like the care you receive from the VA (name specific programs they named, i.e. MFH, HBPC)?
      i. What are the advantages/ disadvantages of these services/programs?

2. How long have you lived in this area?

3. Do you live alone?

   a. If no, who else lives with you?

4. Tell me about what it was like for you during Hurricane Ian.

   a. How was your health been affected by the Hurricane?

   b. What was it like trying to communicate with your family/friends during and after the Hurricane?

   c. Were you able to communicate with your VA healthcare team/providers? How so?

   d. Did the hurricane cause delays in home visits from the VA care team? If so, how long was the delay?

   e. What if any disaster plans or preparation were you aware of that the VA had in place before the hurricane to support you?
      i. How have those changed?

   f. What if any disaster plans did you have in place yourself?
      i. How have those changed?

   g. Have you experienced a disaster like this before? If so, how did Ian compare to previous disasters you experienced?

5. Tell me about your experiences since Hurricane Ian.

   a. What challenges have you faced since the Hurricane?

      i. Health-related?

      ii. Communication?

      iii. Related to coordination with Medical Foster Home coordinator, Home-Based Primary Care team, the VA in general?

      iv. Safety-related?

      v. Other?

   b. What has helped the most during Hurricane recovery?

   c. What resources would have been helpful that you ***did not have*** during and after the Hurricane?

6. Tell me about lessons learned from Hurricane Ian.

   a. How have these lessons influenced the care you receive?

   b. What would you say are the **key things you learned** to be better prepared for a hurricane like Ian in the future?

7. What other things do you feel are important for me to understand about how the VA can best care for Veterans like you during and after a Hurricane?

8. Are there other things you feel are important for me to understand about ***your experience*** during and after the Hurricane?

9. If I have further questions follow-up questions would you be ok with me contacting you later?

10. Finally, is there anyone else you would recommend I talk to on this topic?
