# Supplementary material for: Experiences of Veterans, Caregivers, and VA Home-Based Care Providers before, during, and Post-Hurricane Ian
Source: Geriatrics (Basel). 2024 Jan 10;9(1):10. doi: 10.3390/geriatrics9010010 (PMC10801461; doi:10.3390/geriatrics9010010)
Supplement: Supplementary file 1 [file geriatrics-09-00010-s001.zip › Interview Guide S2 Staff.pdf]

Interview Guide for VA Home Based Long-Term Care Staff, i.e. Medical Foster Home/Home Based Primary Care/Redefining Elder Care in America Program Staff following Hurricane Ian

1. Tell me about your role at your VA.

   a. How long have you served in that role?
      i. How long have you worked at the VA?

   b. Tell me about any other previous experience you have providing home-based care or working with long-term care populations.

2. Tell me about taking care of Veterans during Hurricane Ian.

   a. How did you communicate with Veterans and their caregivers or their families during and after the Hurricane?

   b. How have Veterans and their caregivers fared since the Hurricane?

   c. How have you worked with/supported caregivers/Veterans/Veterans' families since the hurricane?

3. Tell me about your own experiences during and after the hurricane.

   a. Biggest challenges?

   b. What has helped you the most during recovery from the hurricane?

   c. What resources would have been helpful that you did not have?

   d. Have you experienced a disaster like this before? If so tell me about that experience.

4. Tell me about disaster plans or policies in place at your VA before the hurricane to care for Veterans in your VA program who are mostly homebound.

   a. Who was involved in creating these plans or policies?

   b. What training have you received around these plans or policies?

5. Please describe how the disaster plans were implemented during and after the hurricane.

   a. What went well?

   b. What were some of the challenges?

   c. What would you have liked to see improve?

   d. How have any disaster-related plans or policies changed around preparing for and recovering from disasters?
      i. If they have not, would you like them to? How so?

6. Tell me about any training provided to caregivers and/or Veterans in disaster preparedness.

   a. What, if any, other resources related to disaster preparedness are caregivers provided from the VA?

b. What, if any, additional disaster preparedness efforts are you aware of that caregivers or Veterans have in place since Hurricane Ian?

7. Tell me about lessons learned from the hurricane.

8. What would you say are the key ingredients to successful disaster preparedness and recovery related to your program or similar long-term care programs?

9. What other things do you feel are important for me to understand about disaster preparedness or recovery when caring for Veterans like those in your program who have long-term care needs and are mostly homebound?

10. If I have further questions follow-up questions would you be ok with me contacting you later?

11. Finally, is there anyone else you would recommend I talk to on this topic?
