# Supplementary material for: Experiences of Veterans, Caregivers, and VA Home-Based Care Providers before, during, and Post-Hurricane Ian
Source: Geriatrics (Basel). 2024 Jan 10;9(1):10. doi: 10.3390/geriatrics9010010 (PMC10801461; doi:10.3390/geriatrics9010010)
Supplement: Supplementary file 1 [file geriatrics-09-00010-s001.zip › Interview Guide S3 Caregiver.pdf]

Interview Guide for caregivers of Veterans receiving Home Based Primary Care or Medical Foster Home Care before, during, and after Hurricane Ian

1. Tell me about what types of care the Veteran you care for receives from the VA.

    a. *Probes*
        i. Tell me more about what it is like for them to receive that care (specifically probe on whether the Veteran receives care from the VA Medical Foster Home program, or the VA Home Based Primary Care program, or other VA long-term services and supports they may get, like home health)
        ii. Does the Veteran you care for have most of their healthcare appointments at home or in the clinic?
            a. If in the clinic, how do they get to your healthcare appointments?
            b. Tell us more about how you provide care for the Veteran (i.e. help with your care, getting to your appointments, or their medications)?

    b. How long have has the Veteran you care for received care from the VA?

        i. How long has the Veteran you care for received care from the specific programs you mentioned (i.e. MFH program or the HBPC program or home health)?

        ii. How did they decide to receive these types of care (i.e. from MFH or HBPC or home health, etc.)?

    c. How do you like the care the Veteran you care for receives from the VA (name specific programs they named, i.e. MFH, HBPC)?
        i. What are the advantages/ disadvantages of these services/programs?

2. How long have you lived in this area?

3. Do you live with the Veteran?

    a. If no, who else lives with the Veteran, or do they live alone?

4. Tell me about what it was like for you and the Veteran you care for during Hurricane Ian.

    a. What was it like trying to communicate with your family/friends during and after the Hurricane?

    b. Were you able to communicate with your Veteran's VA healthcare team/providers? How so?

    c. Did the hurricane cause delays in home visits from the VA care team? If so, how long was the delay?
    d. What if any disaster plans or preparation were you aware of that the VA had in place before the hurricane to support you and the Veteran you care for?
        i. How have those changed?

    e. What if any disaster plans did you have in place?
        i. How have those changed?

    f. Have you experienced a disaster like this before? If so, how did Ian compare to previous disasters you experienced?

5. Tell me about your experiences since Hurricane Ian.

   a. How has the Veteran you care for's health been affected by the Hurricane?

   b. What challenges have you and the Veteran you care for faced since the Hurricane?

      i. Health-related?

      ii. Communication?

      iii. Related to coordination with Medical Foster Home coordinator, Home-Based Primary Care team, the VA in general?

      iv. Safety-related?

      v. Other?

   c. What has helped the most during Hurricane recovery?

   d. What resources would have been helpful that you ***did not have*** during and after the Hurricane?

6. Tell me about lessons learned from Hurricane Ian.

   a. How have these lessons influenced the care your Veteran receives?

   b. What would you say are the **key things you learned** to be better prepared for a hurricane like Ian in the future?

   c. If you could design the ideal disaster preparedness plan/policies for Veterans like the Veteran you care for, what would they look like?

7. What other things do you feel are important for me to understand about how the VA can best support caregivers like you and older Veterans during and after a Hurricane?

8. Are there other things you feel are important for me to understand about ***your experience*** during and after the Hurricane?

9. If I have further questions follow-up questions would you be ok with me contacting you later?

10. Finally, is there anyone else you would recommend I talk to on this topic?
